# Identifying Profiles and Symptoms of Patients With Long COVID in France: Data Mining Infodemiology Study Based on Social Media

**DOI:** 10.2196/39849

**Published:** 2022-11-22

**Authors:** Amélia Déguilhem, Joelle Malaab, Manissa Talmatkadi, Simon Renner, Pierre Foulquié, Guy Fagherazzi, Paul Loussikian, Tom Marty, Adel Mebarki, Nathalie Texier, Stephane Schuck

**Affiliations:** 1 Kap Code Paris France; 2 Deep Digital Phenotyping Research Unit, Department of Precision Health, Luxembourg Institute of Health Strassen Luxembourg

**Keywords:** long COVID, social media, Long Haulers, difficulties encountered, symptoms, infodemiology study, infodemiology, symptoms, COVID-19, patient-reported outcomes, persistent, condition, topics, discussion, social media, content

## Abstract

**Background:**

Long COVID—a condition with persistent symptoms post COVID-19 infection—is the first illness arising from social media. In France, the French hashtag #ApresJ20 described symptoms persisting longer than 20 days after contracting COVID-19. Faced with a lack of recognition from medical and official entities, patients formed communities on social media and described their symptoms as long-lasting, fluctuating, and multisystemic. While many studies on long COVID relied on traditional research methods with lengthy processes, social media offers a foundation for large-scale studies with a fast-flowing outburst of data.

**Objective:**

We aimed to identify and analyze Long Haulers’ main reported symptoms, symptom co-occurrences, topics of discussion, difficulties encountered, and patient profiles.

**Methods:**

Data were extracted based on a list of pertinent keywords from public sites (eg, Twitter) and health-related forums (eg, Doctissimo). Reported symptoms were identified via the MedDRA dictionary, displayed per the volume of posts mentioning them, and aggregated at the user level. Associations were assessed by computing co-occurrences in users’ messages, as pairs of preferred terms. Discussion topics were analyzed using the Biterm Topic Modeling; difficulties and unmet needs were explored manually. To identify patient profiles in relation to their symptoms, each preferred term’s total was used to create user-level hierarchal clusters.

**Results:**

Between January 1, 2020, and August 10, 2021, overall, 15,364 messages were identified as originating from 6494 patients of long COVID or their caregivers. Our analyses revealed 3 major symptom co-occurrences: asthenia-dyspnea (102/289, 35.3%), asthenia-anxiety (65/289, 22.5%), and asthenia-headaches (50/289, 17.3%). The main reported difficulties were symptom management (150/424, 35.4% of messages), psychological impact (64/424,15.1%), significant pain (51/424, 12.0%), deterioration in general well-being (52/424, 12.3%), and impact on daily and professional life (40/424, 9.4% and 34/424, 8.0% of messages, respectively). We identified 3 profiles of patients in relation to their symptoms: profile A (n=406 patients) reported exclusively an asthenia symptom; profile B (n=129) expressed anxiety (n=129, 100%), asthenia (n=28, 21.7%), dyspnea (n=15, 11.6%), and ageusia (n=3, 2.3%); and profile C (n=141) described dyspnea (n=141, 100%), and asthenia (n=45, 31.9%). Approximately 49.1% of users (79/161) continued expressing symptoms after more than 3 months post infection, and 20.5% (33/161) after 1 year.

**Conclusions:**

Long COVID is a lingering condition that affects people worldwide, physically and psychologically. It impacts Long Haulers’ quality of life, everyday tasks, and professional activities. Social media played an undeniable role in raising and delivering Long Haulers’ voices and can potentially rapidly provide large volumes of valuable patient-reported information. Since long COVID was a self-titled condition by patients themselves via social media, it is imperative to continuously include their perspectives in related research. Our results can help design patient-centric instruments to be further used in clinical practice to better capture meaningful dimensions of long COVID.

## Introduction

Long COVID, also known as postacute sequelae of COVID-19, is one of the many repercussions of the COVID-19 pandemic. Patients who once had COVID-19 and experienced lasting symptoms referred to their condition as “long COVID” and themselves as “Long Haulers” [1]. Long COVID is defined as a persistence of symptoms for several weeks after the onset of COVID-19, with over 20% of those afflicted with it describing them after at least 4 weeks, and over 10% of patients after 3 months [2]. Early in the course of the health crisis, scientists focused on studying the novel SARS-CoV-2 and officials rushed to contain the spread of contamination, paying less attention to long-term effects. While infections were once thought of as short-term, in many cases, they became a lingering illness. The exact prevalence of long COVID is yet to be determined. A meta-analysis by Chen et al [3] estimated that 43% of COVID-19–positive individuals have had long COVID, and an even higher proportion for those who were hospitalized during the acute phase of infection [4]. Patients described their symptoms as long-lasting, fluctuating, and multisystemic, most frequently reporting generalized fatigue [5], respiratory ailments [6], neurological and cardiothoracic disorders, and a partial or complete loss of smell and taste [7].

Long COVID is the first illness arising from social media: the original long COVID hashtag (#LongCovid) appeared on Twitter in May 2020 to illustrate a lengthier and more complex course of the disease than described in the early reports from Wuhan, China [1]. The French hashtag #ApresJ20, which translates to “after day 20,” described symptoms persisting longer than 20 days after contracting the infection. Patient-led groups on social media swiftly assembled, growing into a hub for information-sharing, support, encouragement, and communication among Long Haulers. In just a few months, discussions about long COVID moved from patients, via various media, to formal clinical and policy entities [1]. This highlighted the role of social media in drawing attention to a condition originally deemed invisible or nonexistent.

Indeed, social media has become an integral part of people’s lives over the years. In 2021, the International Telecommunication Union estimated that 4.9 billion people were using the internet [8]. During the COVID-19 pandemic, social media turned into the main source of communication during lockdown [9], witnessing a 17% increase in internet users [8]. Social media has also demonstrated an increasing presence in health care: a rising number of patients have turned to the internet for health-related reasons [10]. Recently, the rise of social media prompted the emergence of infodemiological studies. Studies using data from web-based platforms have proven effective in research, notably in studying influenza-related topics [11-15], HIV/AIDS [16,17], and measles [18]. These studies use data obtained directly from the patient, avoiding lengthy traditional research methods and clinical studies. These especially proved to be useful during the pandemic, as scientists rapidly needed information about the novel coronavirus, and lockdown and social distancing measures disrupted the world. As long COVID rapidly gained awareness owing to social media, one cannot deny the substantial volume of data and respondents on web-based platforms and their impact to ultimately influence public policies. Indeed, there exists a window of opportunity regarding social media as tools for health research [19-22].

While research on long COVID has expanded, many studies relied on web-based surveys or clinical settings [7,23-26]. A limitation of these methods is the lengthy process required to launch the studies, obtain results, and finally to publish them. Social listening, however, offers a foundation for large-scale studies with a fast-flowing outburst of data and the opportunity to listen to patients in real time. The duration of our research spanned 587 days; this will allow us to fill any knowledge gaps that exist beyond the 1-year postinfection mark, ultimately preventing a shortfall in health care's potential during this crisis. Furthermore, this study encompassed multiple social media sources (ie, Twitter, Reddit, Doctissimo, Facebook, and other forums), thus increasing its exhaustivity and possibly inclusivity and representativity.

In this study, we aimed to examine patient feedback on social media regarding their experience with long COVID, using data mining methods. Our aim was to examine the impact of long COVID on Long Haulers by analyzing their main topics of discussion, the difficulties they encountered, and their most reported symptoms.

## Methods

### Study Design and Population

This observational, retrospective, real-world study encompasses data retrieved from social media posts of individuals with long COVID symptoms and their caregivers. The duration of the study spanned from January 1, 2020, to August 10, 2021.

### Data Extraction

Messages written in French, geolocated in France, and posted between January 1, 2020, and August 10, 2021, were included. The data ultimately retrieved are composed of messages from public websites (eg, Twitter) and health-related forums (eg, Doctissimo). Owing to restricted data access and closed groups, only 2 Facebook pages “AprèsJ20” and “Collectif de Malades Covid 19 au Long Cours” were analyzed, while Instagram and WhatsApp were excluded from this study. Keywords associated with long COVID were identified (eg, “long covid,” “chronic covid,” “persistent covid,” “long term covid,” “covid” + “months,” and “covid” + “brain fog”) and subsequently inserted in the extraction query.

Data extraction was performed by the Brandwatch extractor (Cision Ltd). First, we collected publicly available posts found on Twitter and forums featuring one of the relevant keywords. In parallel, we performed web crawling—or data collection—on the previously selected, publicly accessed Facebook pages. Posts were retrieved along with their associated metadata (eg, author or publication date). In this study, there was no distinction in the treatment of posts from the different platforms.

Preprocessing consisted of selecting only relevant messages based on several exclusion criteria: posts of 5 words or less and those exceeding 10,000 characters were excluded, as they are typically found to be irrelevant. Duplicates, posts not written in French, and sources deemed unsafe or inapplicable to our study (eg, advertising websites and forums related to cars, pets, or animals) were also excluded.

To further advance the filtering process, a supervised machine learning algorithm was applied to identify posts associated with patients’ or caregivers’ experiences. This algorithm was previously developed using a training set of 12,330 messages related to different health domains (dermatology, tobacco use, and oncology, among others). The method consists of a pipeline featuring 2 XGBoost [27] classifiers (one for caregivers’ experiences and one for patients’ experiences) applied successively. This method allowed us to identify if a post belonged to a patient, a caregiver, or neither. Both classifiers are based on features combining pronouns and lexical fields describing relatives and pathologies (eg, “my [pronoun] father [relative] has cancer [pathology]”). We trained the algorithm by first identifying the caregivers; this was carried out on the whole data set. To determine patients’ messages, we then reapplied the algorithm on the rest of the data set (excluding the already identified caregiver messages). Evaluation of performances yielded F1-scores (a measure of accuracy combining precision and recall) of 88.0% and 87.0% for the caregiver and patient classifier, respectively.

In this study, to assess its performance on COVID-19–related data, the algorithm’s performances were measured on a random sample of 700 messages classified as patients’ or caregivers’ messages. The pipeline predictions were then used to filter out posts that do not describe personal experiences with long COVID.

In this study, only posts from patients and caregivers were considered for analysis.

### Ethical Considerations

This study included data from publicly available sources; private groups or web pages were thus excluded from our data extraction process. We did not seek approval as users automatically grant their consent for the reuse of their data when they post on public platforms. Furthermore, the results of this study do not contain any identifiable information and are presented in aggregate. Information such as the name, username or handle, geographic locations, or any other sensitive data were not included.

### Data Analysis

#### Topics of Discussion

Main discussion themes were identified through the examination of all 15,364 posts from patients and caregivers regarding long COVID. This was performed using Biterm Topic Modeling (BTM) with the BTM R package [28]. BTM is a natural language processing–based text mining approach, which clusters similar texts on the basis of common discussion topics and provides lists of words to be interpreted for cluster labeling [29]. Topic modeling considers documents (messages and posts) as a mixture of topics that are a probability distribution over the words of the data set. A post can then be associated to its most prominent topic. BTM provides, for each topic, a list of the 20 highest-probability words and all the posts associated with the topic. Through human interpretation, these lists of words were then used to label the topics, and the associated posts were thoroughly scanned to ensure correct interpretation.

#### Unmet Needs and Difficulties Encountered

A manual review of posts identified the unmet needs described by patients with long COVID and their caregivers. A total of 450 messages were randomly selected from 3 types of sources: Twitter, forums, and selected public Facebook pages (n=150 messages from each type of source). We considered that this sample was sufficient and reasonable to have an overall view of the different types of unmet needs. To identify the unmet needs and difficulties of patients and caregivers, independent evaluators reviewed this sample via qualitative analysis: difficulties were coded in accordance with a previously set grid of categories to guarantee standardized data labeling and depending on whether the difficulty pertained to a patient, caregiver, or both. The categorization process was not mutually exclusive: the same message could contain multiple difficulties.

#### Reported Symptoms

To identify patients’ and caregivers’ reported symptoms, the data set resulting from preprocessing cleaning was analyzed using the MedDRA dictionary. The MedDRA dictionary is governed by a 5-level structure of hierarchy: a system organ class (SOC) is the highest or most general level, which is further subdivided into high-level group terms, high-level terms, preferred terms (PTs), and the most detailed lowest-level terms (LLTs). This last level was used for the detection of reported symptoms to achieve maximal thoroughness [30-32]. Since the MedDRA dictionary lacked terms related to long COVID at the time of the study, we manually added to it a list of symptoms identified through literature review [5-7]. All these terms were then searched in the messages and sorted in accordance with their frequency of occurrence, which allowed us to create a list of PTs of interest by selecting the most recurrent and relevant PT. The last step consisted of manual cleaning of the LLTs associated with the list of PTs, and pooling similar PTs (eg, fatigue and asthenia). Results were then sorted in accordance with the frequency at which they were reported with the top 15 PTs selected for this study. A manual review was then performed to assess whether the medical concepts of those PTs were indeed long COVID symptoms. Once achieved, the set of LLTs associated with the selected PTs was used for detecting symptoms. Hereinafter, we shall refer to the top 15 PTs as “symptoms’ PTs.”

#### Co-occurrences and Standard Profile of a Patient With Long COVID in Terms of Symptoms

Associations were assessed by computing co-occurrences in users’ posts as pairs of PTs. Counts of each PT in the total of their posted content was used to cluster users through hierarchical clustering. Users who mentioned at least 2 different symptoms’ PTs in their messages were considered (n=289). A heat map was created to clearly display the significant co-occurrences.

Regarding the standard profile of a patient with long COVID, symptoms were displayed in accordance with the volume of posts mentioning them and then aggregated at the user level.

### Chronological Monitoring of Symptoms

The evolution of symptoms was monitored through time: we collected messages pertaining to users who had written a minimum of 5 posts and featuring the selected symptoms’ PTs within 18 months of their infection dates. We selected a threshold of a minimum of 5 messages from the same user to have enough data to follow their symptoms over time.

For the selected users, using regular expressions of duration and dates (eg, “It has been six months” and “since April”) helped determine the estimated date of the COVID-19 infection. Following this information, a manual review of the messages allowed the analysis of symptom evolution per quarter year.

## Results

### Population and Posts

**Figure 1:**
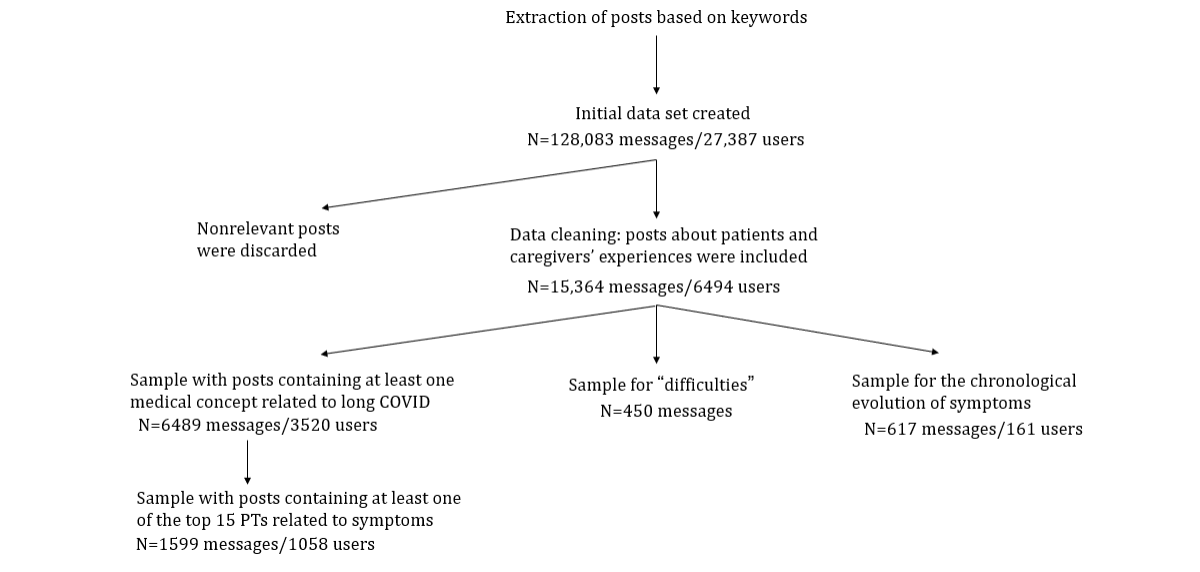
Flow chart of the data cleaning and sample selection processes. PT: preferred term.

Between January 1, 2020, and August 10, 2021, a total of 128,083 messages were retrieved, which were written by 27,387 French speakers in France, ranging from journalists, politicians, citizens, or individuals recently infected with COVID-19 fearing a long-term progression of symptoms. A total of 21 sources were included in this study; however, the majority of the retrieved data (121,560/128,083, 94.9%) were found on Twitter. Subsequent analyses were not segmented among sources. As previously mentioned, a machine learning algorithm was applied to identify posts associated with patients or caregivers’ experiences. As a result, of the 128,083 retrieved messages, 15,364 messages were identified as having originated from 6494 patients with long COVID or their caregivers (Figure 1).

The patient-caregiver algorithm was evaluated on long COVID data through a manual review of a random sample of 700 messages. Comparing pipeline predictions to manual coding yielded the following performance results: an accuracy of 87.5%, F1-score of 89.7%, sensibility of 96.3%, specificity of 75.8%, and a precision of 84.0%. Twitter remained the main source of expression with 93.8% (14,410/15,364) of messages.

Our analysis revealed that the first mention of “covid chronique” (which translates to “chronic COVID-19”) appeared on social media on March 16, 2020. Less than a month later on April 12, 2020, the hashtag #apresJ20 was first mentioned on Twitter in the following message (translated from French): “Lack of information for people who continue to have symptoms after D20. It would be nice to share our feelings and feel less alone so I'm opening this poll for those who are still struggling after D20 #COVID19 #afterD20.”

**Figure 2:**
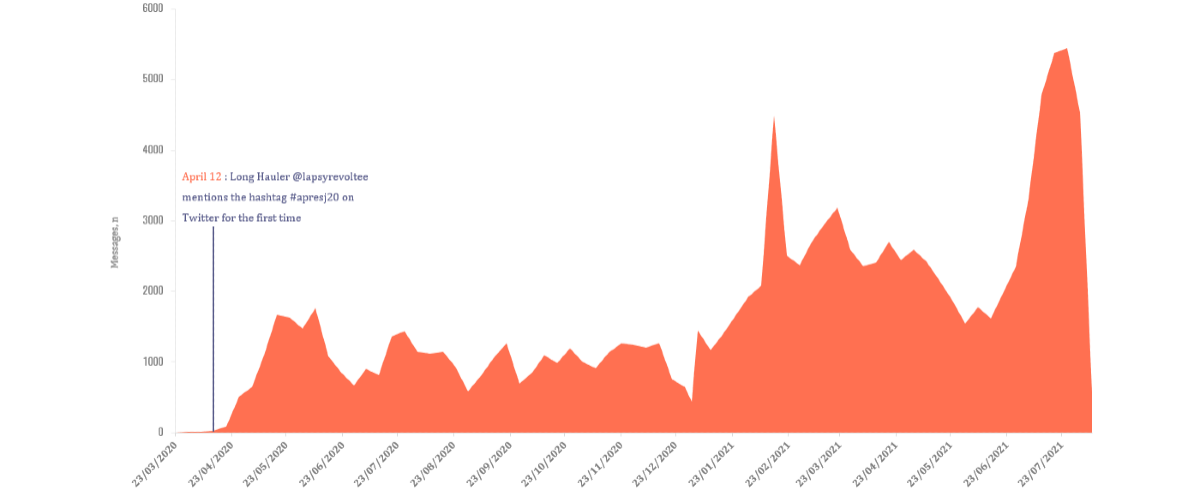
Evolution of the percentages of number of messages per main topic between March 2020 and July 2021.

Following the introduction of the hashtag #ApresJ20, it rapidly went viral in France; Twitter and Facebook witnessed a rise in the number of users sharing their experiences with long COVID, particularly after the launch of pages and groups dedicated to this subject (Figure 2).

### Data Analysis

#### Topics of Discussion

**Figure 3:**
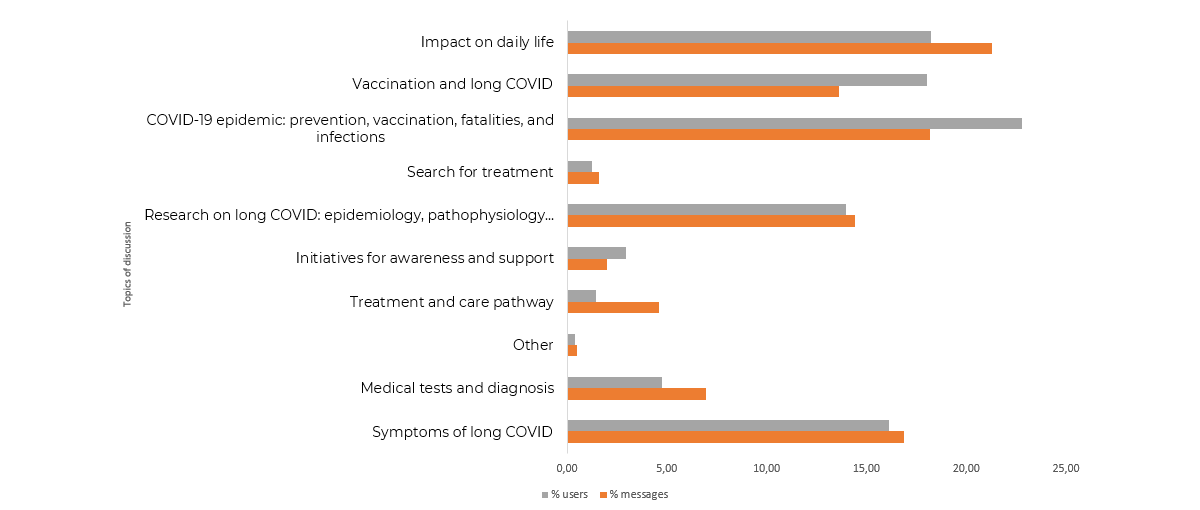
Proportions of messages and internet users with the main discussion topics.

Following the application of the BTM on the data set including all the analyzed forums (Multimedia Appendix 1), various discussion topics were identified through human interpretation of each topic’s most associated terms. For instance, “vaccine,” “protect,” and “long” were among the top terms of the topic “Vaccination and Long Covid” after translation. The main revealed topics are featured in Figure 3.

The 5 primary topics of discussion centered around the COVID-19 pandemic in general (2793/15,364, 18.2% of messages) in addition to issues related to long COVID were as follows: impact on daily life (3269/15,364, 21.3%), reported symptoms (2592/15,364, 16.9%), vaccination (2090/15,364, 13.6%), and research (2212/15,364, 14.4%).

The topic “Covid-19 pandemic” was discussed by the highest number of users (1480/6494, 22.8%), while the topic “impact on daily life” received the largest volume of posts (3269/15,364, 21.3%).

**Figure 4:**
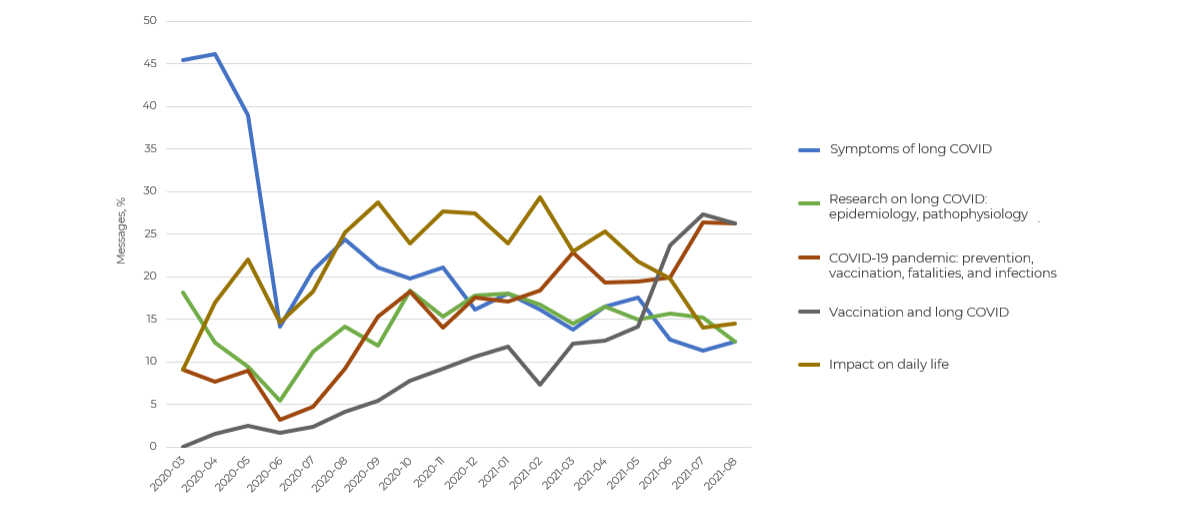
Chronological evolution of the main discussion topics by the proportion of messages.

The monitoring of these 5 topics through time revealed a peak in the number of posts around the subject of “symptoms” in the first half of 2020 (5/11, 45.5% of the messages posted in March 2020 about long COVID; Figure 4).

Furthermore, the topic “COVID-19 pandemic” progressively gained momentum and was increasingly discussed over time after the second half of 2020 (31/982, 3.2% of the messages posted in June 2020, ending at 204/775, 26.3% of the messages posted in August 2021; Figure 4). In contrast, discussions around the impact on daily life gradually decreased. On completion of this study, the topics of “vaccination” (204/775, 26.3%) and “COVID-19 pandemic” (204/775, 26.3%) were the most frequently discussed among users (Figure 4).

#### Unmet Needs and Difficulties Encountered

Of the 450 messages analyzed, 424 included at least 1 difficulty reported by a patient or caregiver. These messages contained a total of 709 difficulties, which were sorted into 34 categories overall, with a single message possibly containing more than 1 category of difficulties. The top 20 categories of the main reported difficulties are featured in Table 1.

The main difficulties reported by patients in relation to long COVID were the management of their symptoms (150/424, 35.4% of messages), which were described as diverse, lasting several weeks or months, fluctuating over time, and involving relapses. Patients also reported a psychological impact characterized by a fear of the unknown (64/424, 15.1%). Additionally, messages mentioned a feeling of pain (51/424, 12.0%) and a deterioration in general well-being (52/424, 12.3%), particularly owing to intense and chronic fatigue. The impact on daily and professional life, mentioned in 9.4% (40/424) and 8.0% (34/424) of messages, respectively, was described by patients and caregivers as considerably reducing their quality of life. Furthermore, 13.7% (58/424) of the reported difficulties were centered around the lack of recognition of long COVID by health care providers, public and health authorities, or even patients’ close circles. In fact, several patients reported that doctors were questioning the clinical validity of their symptoms, and, in some cases, even suggesting that the problems were simply due to stress. A patient detailed her experience with a health care professional in the following message (translated from French):

Doctors have a hard time diagnosing long Covid... Some have told me that I was in denial about being pregnant (I haven't had intercourse in several months), or that I was starting menopause (not true after blood work), that it was depression or that it was all in my head! A shame that this disease is still not recognized nor treated

**Table 1:** Proportions of messages featuring the main reported difficulties.

Difficulties	Messages, n (%)
Concern with and management of symptoms of long COVID	150 (35.4)
Psychological impact of long COVID on patients and the stress of uncertainty	64 (15.1)
Lack of recognition of long COVID	58 (13.7)
Alteration of the general state of health with chronic fatigue, loss of capacity, brain fog, etc	52 (12.3)
Management of pain	51 (12.0)
Impact on daily activities	40 (9.4)
Professional impact for the patient: part-time work, absences, etc	34 (8.0)
Patient dissatisfaction with the provision of care	34 (8.0)
Fears and management of aggravations and relapses of long COVID	33 (7.9)
Difficulty in accessing care: long waiting time, difficulty taking an appointment, lack of experts on the subject, etc	29 (6.8)
Issues related to the lack of training of health care personnel on long COVID	17 (4.0)
Sequelae of COVID-19	17 (4.0)
Sharing, experiences, and support: discussion groups, social networks, etc	16 (3.8)
Worried or concerned about the future, life expectancy, or difficulty planning ahead	16 (3.8)
Communication and relationship problems: lack of empathy, conveyance of information, medical jargon, etc	11 (2.6)
Disagreement in health management: heterogeneity of medical decisions and opinions, disagreement between the patient and medical team, etc	10 (2.4)
Lack of general knowledge or scientific information about long COVID	9 (2.1)
Multiple treatment failure or ineffective treatments	9 (2.1)
Financial impact of health care for patients	9 (2.1)
Impact of long COVID on the management of comorbidities	7 (1.7)

#### Reported Symptoms

Overall, 6489 messages posted by 3520 users in the different forums had expressed at least 1 medical concept related to long COVID. The most reported symptoms were revealed on the basis of pooled PTs, with 1599 messages written by 1058 users having expressed at least one of the top 15 PTs. An evaluation of noise (ie, random data errors) on a random sample of 400 messages revealed an 86.0% correct classification, meaning that 86.0% of PTs actually corresponded to symptoms of long COVID. Additionally, an assessment was performed on 10 random messages for each PT, yielding a correct classification of 90.2% on average.

Medical concepts were also categorized in accordance with the MedDRA dictionary’s SOC based on 7 organ categories: systemic, respiratory, nervous, psychiatric, musculoskeletal, cardiac, and gastrointestinal. The majority of messages (995/1599, 62.2%) pertained to the “systemic” category (Multimedia Appendix 2).

In addition, health ailments were related to patients’ respiratory system (267/1599, 16.7%), nervous system (264/1599, 16.5%), and psychiatric system (252/1599, 15.8%; Multimedia Appendix 2).

The pooling of PTs revealed a range of symptoms related to long COVID with the top 3 most reported ones in patients’ and caregivers’ messages being asthenia (835/1599, 52.2% of messages), dyspnea (267/1599, 16.7%), and anxiety (242/1599, 15.1%; Multimedia Appendix 3).

Indeed, patients reported a feeling of chronic fatigue and weakness, as described in the following message (translated from French): “I had so much vital energy that I tired everyone around me! Ever since my long Covid, I remain confined, low blood pressure, insane fatigue, seizures of all kinds, yes, I have no more energy!”

#### Co-occurrences and Standard Profile of a Patient With Long COVID in Terms of Symptoms

**Figure 5:**
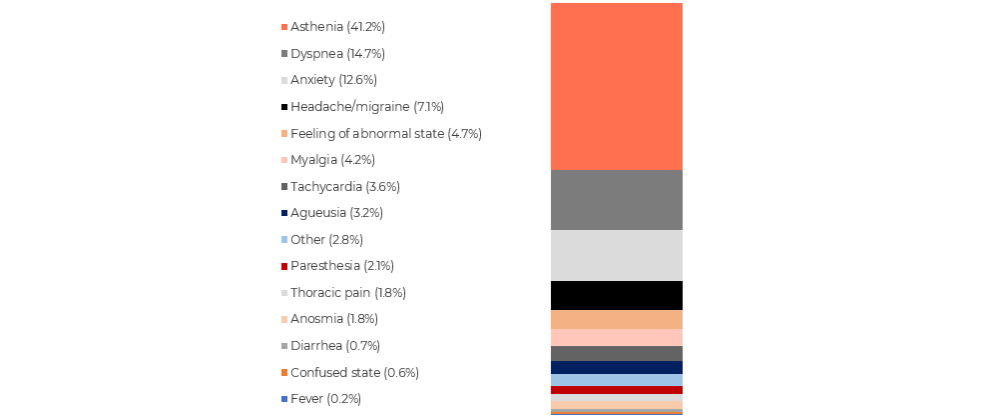
Distribution of reported symptoms related to an individual.

Among the patients who described a symptom from among the top 15 symptoms (1584 patients), 41.2% (n=652) of them experienced asthenia, 14.7% (n=233) reported dyspnea, and 12.6% (n=200) experienced anxiety (Figure 5).

**Figure 6:**
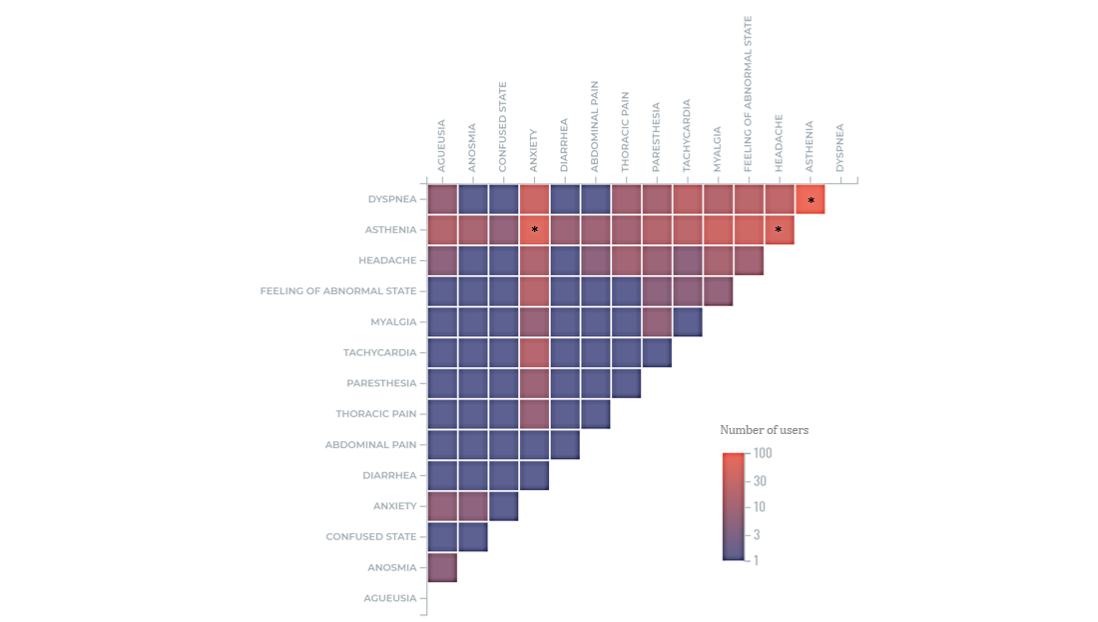
Heat map of the co-occurrences that appeared at least 5 times.

Several symptoms were also frequently and simultaneously reported by patients or caregivers. Users who mentioned at least 2 different symptoms’ PTs in their messages were considered (n=289). A heat map featuring the co-occurrences that appeared at least 5 times is displayed as a logarithmic scale in Figure 6. The highest proportion of patients (102/289, 35.3%) reported asthenia paired with dyspnea, followed by 22.5% (65/289) of patients who experienced asthenia along with anxiety, while 17.3% (50/289) of patients experienced asthenia in combination with headaches (Figure 5).

Furthermore, the “clustering” method allowed the identification of the 3 main standard profiles of patients in relation to their symptoms: profile A (n=406 patients) reported exclusively 1 symptom of asthenia; profile B (n=129) expressed anxiety (n=129, 100%), asthenia (n=28, 21.7%), dyspnea (n=15, 11.6%), ageusia or loss of a sense of taste (n=3, 2.3%); and finally, profile C (n=141) described dyspnea (n=141, 100%) and asthenia (n=45, 31.9%).

#### Chronological Monitoring of Symptoms

To monitor the evolution of symptoms through time, the initial data set was filtered to retain users with enough content to be followed: of the 15,364 messages in the initial data set, 3062 posts by 1493 users included a regular expression of duration and dates. Among those users, 330 had posted at least 5 messages; their posts amounted to a total of 1765, including 712 posts (from 217 users) with at least 1 mention of a PT. Finally, we retained 617 posts by 161 users featuring PTs related to symptoms within 18 months of their infection dates. This final data set of messages revealed the following reported symptoms: asthenia, dyspnea, headache, a feeling of abnormal state, and myalgia.

**Figure 7:**
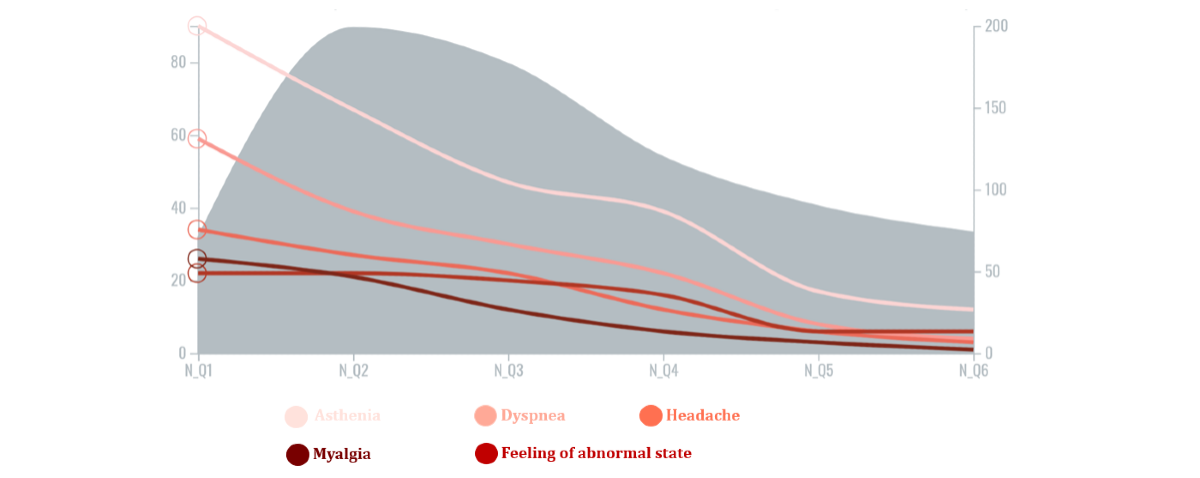
Evolution of the number of occurrences per symptom and the number of messages with a mention of a symptom per quarter year.

Our analysis showed a peak in the number of messages in the second trimester, followed by a gradual decrease over time (Figure 7). Furthermore, 79 of 161 (49.1%) users continued expressing symptoms between 3 months and 6 months post infection, 32.9% (53/161) between 9 and 12 months, and 20.5% (33/161) between 15 and 18 months. 

## Discussion

### Principal Findings

#### Background

This study revealed—through the lens of Long Haulers—the multifaceted challenges and repercussions associated with long COVID. The main topics of discussion on social media centered around the impact on daily life, reported symptoms, and vaccination. Patients expressed difficulties related to the management of their symptoms, the impact on their mental health, and the impact on their daily and professional lives. Our analyses revealed 3 major symptom co-occurrences: asthenia-dyspnea, asthenia-anxiety, and asthenia-headaches. We identified 3 profiles of patients in relation to their symptoms: profiles A, B, and C, which mainly reported asthenia, anxiety, and dyspnea, respectively. Approximately 49.1% of users (79/161) continued expressing symptoms after more than 3 months post infection, and 20.5% (33/161) after 1 year.

#### Role of Social Media

The COVID-19 pandemic gained momentum on social media. Met with skepticism from medical professionals, coined as “medical gaslighting” [33,34], Long Haulers turned to web-based platforms, mainly Twitter (93.8% of total messages), to share their experiences. In fact, many infodemiological studies have documented the popularity of Twitter among internet users and used it as the main source of data [20,35-38].

Our study showed multiple peaks in communication coinciding with notable events. The #ApresJ20 launched a nationwide discussion on long COVID, with a peak in the number of messages soon after the hashtag was first mentioned in April 2020. Other peaks later followed in the course of our study in response to various events: in October 2020, after the Apresj20-Association Covid Long France launched along with its website, Facebook, and Twitter pages, offering a support group, patient experiences, resources, and information regarding long COVID [39]; in February 2021, after the association proposed resolutions to the National Assembly for the recognition of individuals with long COVID; and in July 2021, after President Emmanuel Macron extended the health pass and announced mandatory vaccination for certain professions [40]. This further demonstrated the role that social media played in propelling long COVID and echoing the voices of Long Haulers during the health crisis; it also showed that communication on social media mirrored notable events related to long COVID.

#### Topics of Discussion and Difficulties Encountered

Not only was social media effective for collecting substantial volumes of data, but also it communicated patients’ sentiments, perceptions, and pain points. Patients reported physical and psychological sequelae that affected their day-to-day lives, complained of the heavy toll they were experiencing post COVID-19 infection, and criticized the quality of care afforded to them. Health providers’ lack of knowledge of long COVID have led to serial misdiagnoses, and patients felt “invisible,” uncertain if they will ever be cured of their physical pain, deteriorating well-being, and chronic fatigue.

Our analyses revealed that posts about “symptoms” initially dominated discussion topics, which is consistent with the findings of other infodemiological studies [21,41]. As the condition was relatively unknown in the first semester of 2020, social media served as a medium for patients and caregivers to relate to people with similar symptoms, thus creating a community for support and communication. A lack of information, recognition, and acknowledgment were the main catalysts behind the rallying forces of advocacy groups on social media; this prompted official entities, in the latter half of the year, to officially recognize long COVID and offer information and support for those afflicted with it [42]. Main topics of discussion also revolved around its impact on quality of life. Patients shared their experiences with long COVID and its impact on their everyday activities: mobility, housework, sports, etc. According to a study by Shah et al [43], survivors of COVID-19 reported a considerable impact of long COVID on their quality of life with problems ranging from physical (eg, limited mobility, disrupted usual activities, pain, and discomfort) to psychological (eg, anxiety, stress, and depression). Interestingly, discussions around the impact of long COVID on daily life gradually decreased, suggesting a lighter burden on some patients as their symptoms improved or as they came to accept their condition as their new health baseline.

Long COVID spread to many aspects of patients’ lives; it affected their professional activities as they felt incapable of resuming work owing to their fragile state of health. Our findings align with those of Davis et al [7], where patients experienced difficulties going back to their work; among those who did, many reported experiencing relapse and could no longer continue their work activities. Faghy et al [44] corroborated these results, with patients reporting reduced health and capacity to participate in daily and work activities. This highlights the importance of officials’ recognition of long COVID as a debilitating condition, hence offering those afflicted with it a proper recovery time before resuming their job. In that case, patients should be able to have access to financial government assistance, flexible work hours, or the possibility of teleworking. A holistic approach to restore their pre–COVID-19 health and quality of life, tackling the numerous and multifaceted challenges that patients have highlighted, is also of utmost importance.

An increasingly mentioned topic throughout our study involved “vaccination”; it reached its peak toward the latter half of 2021, coinciding with the implementation of compulsory vaccination to certain professions. This topic triggered debate among Long Haulers: some of them were apprehensive, while others reported the effectiveness of vaccines in alleviating or curing their condition. While the exact pathophysiology of Long COVID is still unknown, evidence shows that getting vaccinated might attenuate symptoms [45].

#### Symptoms and Co-occurrences

Long Haulers have reported symptoms affecting various body organs. The SOC revealed that symptoms mainly pertained to the “systemic” category, followed by the respiratory, nervous, and psychiatric systems. According to Nalbandian et al [46], long COVID also affects the excretory, circulatory, integumentary, and endocrine systems. The main reported symptoms were asthenia and dyspnea, which is consistent with the findings of other studies [5,6]. Additionally, patients described experiencing anxiety due to either the effect of long COVID on brain health or to “medical gaslighting.” A study by Taquet et al [47] on neurological and psychiatric sequelae in survivors of COVID-19 revealed similar results, as patients reported experiencing anxiety even 6 months post COVID-19 infection.

The map of co-occurrences revealed the most commonly reported symptoms that collocated, and solidified the results obtained in the SOC. It revealed 3 major co-occurrences: asthenia (systemic)-dyspnea (respiratory), asthenia-anxiety (psychiatric), asthenia-headaches (nervous). The clustering method further corroborated these findings, as asthenia, anxiety, and dyspnea were found at the top of the 3 main standard profiles of Long Haulers. This raises concern regarding the damage that long COVID may have on organ systems, and highlights the need for a thorough examination of its repercussions. According to Graham et al [48], the main reported symptoms including “brain fog,” persistent fatigue, and depression or anxiety affected Long Haulers’ cognition and quality of life [48].

This study showed a lingering effect of long COVID, with patients still reporting symptoms after 6 months and even after 1 year, albeit to a lesser extent. These findings entail a lasting impact on patients in various aspects of their lives. According to the French e-cohort study ComPaRe [49], among patients who were symptomatic after 2 months, 85% of them reported persisting symptoms 1 year after symptom onset. Another study [50] revealed a prevalent feeling of severe fatigue among patients in long COVID online support groups. Whether they are physical, psychological, social, professional, or financial, the debilitating sequelae must be further explored to achieve better management.

### Limitations

We recognize several limitations related to our study.

First, our research was limited to French-language social media posts and to individuals of certain socioeconomic demographics and literacy capacity, who have access to the internet and are capable and knowledgeable enough to post messages on social media. However, considering that 93% of the French population comprises internet users [51], we may safely assume that our study is adequately representative of the French Long Haulers.

Another limitation is that the majority of our data were obtained from Twitter. However, given that Twitter is one of the most visited websites in France with more than 16 million monthly users, it is no surprise that most of the data used is this study originated from it [52]. This source has specific features, notably a limit in post length and a high reactivity to events. Other sources such as health discussion forums tend to have less but more complete content on a patient’s experience. The disparities among the different sources might have had an impact on some of our analyses such as topic modeling and symptom identification, which are based on content analysis. Although Twitter might have created a bias since it constituted the majority of our data set, Twitter data were essential to our research since the term “long COVID” originated from a tweet [1].

The annotation included a small pool of messages; it might also be prone to the subjective bias of the annotators who performed it. As such, our findings might not be accurately representative of the global population. However, several annotators were involved to limit this bias.

Owing to ethical reasons, our study included only openly accessible web-based networks and, as a result, lacked other platforms with restricted access, such as WhatsApp and Instagram.

### Conclusions

Long COVID is a lingering condition that affects the lives of people worldwide, physically and psychologically. It impacts Long Haulers’ quality of life, everyday tasks, and professional activities. The role of social media in raising and delivering Long Haulers’ voices is undeniable: patients turned to social media to document their negative experiences post COVID-19 infection, search for information regarding their condition, exchange experiences and resources, gain recognition via advocacy groups, and find support in times of uncertainty. It also has the potential to rapidly provide large volume of valuable patient-reported information. Considering the fact that long COVID was a self-titled condition by the patients themselves, it is imperative to continuously include their perspectives in research on long COVID; ignoring this aspect would simply lead to ignoring the key element of how this condition initially emerged. This study provides a good understanding of patients’ perceptions, physical and psychological symptoms, and the difficulties they encountered during their illness. The data presented here can help design patient-centric instruments to be used in clinical practice to better capture meaningful dimensions of long COVID. Further research is imperative to bridge the knowledge gap about long COVID and improve the management of the condition by the health care system.

## Appendix

Multimedia Appendix 1 List of sources.

Multimedia Appendix 2 Proportions of messages with at least 1 occurrence of the system organ class categorized in accordance with the different organ systems.

Multimedia Appendix 3 Proportions of messages featuring at least one occurrence of a PT related to symptoms.

## Abbreviations

BTM: Biterm Topic Modeling
LLT: lowest-level term
PT: preferred term
SOC: system organ class

## References

[1] Callard F, Perego E (2021). How and why patients made long Covid. Soc Sci Med.

[2] (2021). Symptômes prolongés suite à une Covid-19 de l’adulte - Diagnostic et prise en charge. Haute Autorité de Santé.

[3] Chen C, Haupert S, Zimmermann L, Shi X, Fritsche L, Mukherjee B (2022). Global prevalence of post-coronavirus disease 2019 (COVID-19) condition or long COVID: a meta-analysis and systematic review. J Infect Dis.

[4] Ghosn J, Piroth L, Epaulard O, Le Turnier P, Mentré F, Bachelet D, Laouénan C (2021). Persistent COVID-19 symptoms are highly prevalent 6 months after hospitalization: results from a large prospective cohort. Clin Microbiol Infect.

[5] Shah W, Hillman T, Playford ED, Hishmeh L (2021). Managing the long term effects of covid-19: summary of NICE, SIGN, and RCGP rapid guideline. BMJ.

[6] Cares-Marambio K, Montenegro-Jiménez Y, Torres-Castro R, Vera-Uribe R, Torralba Y, Alsina-Restoy X, Vasconcello-Castillo L, Vilaró J (2021). Prevalence of potential respiratory symptoms in survivors of hospital admission after coronavirus disease 2019 (COVID-19): A systematic review and meta-analysis. Chron Respir Dis.

[7] Davis HE, Assaf GS, McCorkell L, Wei H, Low RJ, Re'em Y, Redfield S, Austin JP, Akrami A (2021). Characterizing long COVID in an international cohort: 7 months of symptoms and their impact. EClinicalMedicine.

[8] (2022). Statistics. ITUPP Bucharest.

[9] Pérez-Escoda A, Jiménez-Narros C, Perlado-Lamo-de-Espinosa M, Pedrero-Esteban LM (2020). Social networks' engagement during the COVID-19 pandemic in Spain: health media vs. healthcare professionals. Int J Environ Res Public Health.

[10] Smailhodzic E, Hooijsma W, Boonstra A, Langley DJ (2016). Social media use in healthcare: A systematic review of effects on patients and on their relationship with healthcare professionals. BMC Health Serv Res.

[11] Bour C, Ahne A, Schmitz S, Perchoux C, Dessenne C, Fagherazzi G (2021). The use of social media for health research purposes: scoping review. J Med Internet Res.

[12] Yom-Tov E, Gabrilovich E (2013). Postmarket drug surveillance without trial costs: discovery of adverse drug reactions through large-scale analysis of web search queries. J Med Internet Res.

[13] Seo D, Jo M, Sohn CH, Shin S, Lee J, Yu M, Kim WY, Lim KS, Lee S (2014). Cumulative query method for influenza surveillance using search engine data. J Med Internet Res.

[14] Sharpe JD, Hopkins RS, Cook RL, Striley CW (2016). Evaluating Google, Twitter, and Wikipedia as tools for influenza surveillance using Bayesian change point analysis: a comparative analysis. JMIR Public Health Surveill.

[15] Santos JC, Matos S (2014). Analysing Twitter and web queries for flu trend prediction. Theor Biol Med Model.

[16] Adrover C, Bodnar T, Huang Z, Telenti A, Salathé M (2015). Identifying adverse effects of HIV drug treatment and associated sentiments using Twitter. JMIR Public Health Surveill.

[17] Mavragani A, Ochoa G (2018). Forecasting AIDS prevalence in the United States using online search traffic data. J Big Data.

[18] Mollema L, Harmsen IA, Broekhuizen E, Clijnk R, De Melker H, Paulussen T, Kok G, Ruiter R, Das E (2015). Disease detection or public opinion reflection? Content analysis of tweets, other social media, and online newspapers during the measles outbreak in The Netherlands in 2013. J Med Internet Res.

[19] Sinnenberg L, Buttenheim AM, Padrez K, Mancheno C, Ungar L, Merchant RM (2017). Twitter as a tool for health research: a systematic review. Am J Public Health.

[20] Odlum M, Yoon S, Broadwell P, Brewer R, Kuang D (2018). How Twitter can support the HIV/AIDS response to achieve the 2030 eradication goal: in-depth thematic analysis of world AIDS day tweets. JMIR Public Health Surveill.

[21] Santarossa S, Rapp A, Sardinas S, Hussein J, Ramirez A, Cassidy-Bushrow AE, Cheng P, Yu E (2022). Understanding the #longCOVID and #longhaulers Conversation on Twitter: multimethod study. JMIR Infodemiology.

[22] Jordan S, Hovet S, Fung I, Liang H, Fu K, Tse Z (2018). Using Twitter for public health surveillance from monitoring and prediction to public response. Data.

[23] Nikravesh I (2021). Long COVID can negatively impact physical and cognitive function, employment, and quality of life for at least one year. EurekAlert!.

[24] Taquet M, Dercon Q, Luciano S, Geddes JR, Husain M, Harrison PJ (2021). Incidence, co-occurrence, and evolution of long-COVID features: A 6-month retrospective cohort study of 273,618 survivors of COVID-19. PLoS Med.

[25] Cox T (2021). How many people get 'long COVID?' More than half, researchers find. The Pennsylvania State University.

[26] Yong SJ (2021). Long COVID or post-COVID-19 syndrome: putative pathophysiology, risk factors, and treatments. Infect Dis (Lond).

[27] Chen T, He T, Benesty M (2015). Xgboostxtreme gradient boosting. R package version 04-2.

[28] Yan X Shortext.Github.Io. Github.

[29] Yan X, Guo J, Lan Y, Cheng X (2013). A biterm topic model for short texts.

[30] Abdellaoui R, Schück S, Texier N, Burgun A (2017). Filtering entities to optimize identification of adverse drug reaction from social media: how can the number of words between entities in the messages help?. JMIR Public Health Surveill.

[31] Schäfer F, Faviez C, Voillot P, Foulquié P, Najm M, Jeanne J, Fagherazzi G, Schück S, Le Nevé B (2020). Mapping and modeling of discussions related to gastrointestinal discomfort in French-speaking online forums: results of a 15-year retrospective infodemiology study. J Med Internet Res.

[32] Schück S, Roustamal A, Gedik A, Voillot P, Foulquié P, Penfornis C, Job B (2021). Assessing patient perceptions and experiences of paracetamol in France: infodemiology study using social media data mining. J Med Internet Res.

[33] (2020). Living with Covid19. National Institute for Health and Care Research.

[34] Rubin R (2020). As their numbers grow, COVID-19 "Long Haulers" stump experts. JAMA.

[35] Zeraatkar K, Ahmadi M (2018). Trends of infodemiology studies: a scoping review. Health Info Libr J.

[36] Bian J, Zhao Y, Salloum RG, Guo Y, Wang M, Prosperi M, Zhang H, Du X, Ramirez-Diaz LJ, He Z, Sun Y (2017). Using social media data to understand the impact of promotional information on laypeople's discussions: a case study of Lynch syndrome. J Med Internet Res.

[37] Chen T, Dredze M (2018). Vaccine images on Twitter: analysis of what images are shared. J Med Internet Res.

[38] Hswen Y, Naslund JA, Brownstein JS, Hawkins JB (2018). Monitoring online discussions about suicide among Twitter users with schizophrenia: exploratory study. JMIR Ment Health.

[39] Long COVID France. Long-Covid Europe.

[40] Salvi E (2021). Macron trumpets own record as he announces mandatory vaccines for health staff and Covid 'passports'. Mediapart.

[41] Miyake E, Martin S (2021). Long Covid: online patient narratives, public health communication and vaccine hesitancy. Digit Health.

[42] (2020). WHO Director-General's opening remarks at the media briefing on COVID-19 - 21 August 2020. World Health Organization.

[43] Shah R, Ali FM, Nixon SJ, Ingram JR, Salek SM, Finlay AY (2021). Measuring the impact of COVID-19 on the quality of life of the survivors, partners and family members: a cross-sectional international online survey. BMJ Open.

[44] Faghy MA, Maden-Wilkinson T, Arena R, Copeland RJ, Owen R, Hodgkins H, Willmott A (2022). COVID-19 patients require multi-disciplinary rehabilitation approaches to address persisting symptom profiles and restore pre-COVID quality of life. Expert Rev Respir Med.

[45] Li X, Raventós B, Roel E, Pistillo A, Martinez-Hernandez E, Delmestri A, Reyes C, Strauss V, Prieto-Alhambra D, Burn E, Duarte-Salles T (2022). Association between covid-19 vaccination, SARS-CoV-2 infection, and risk of immune mediated neurological events: population based cohort and self-controlled case series analysis. BMJ.

[46] Nalbandian A, Sehgal K, Gupta A, Madhavan MV, McGroder C, Stevens JS, Cook JR, Nordvig AS, Shalev D, Sehrawat TS, Ahluwalia N, Bikdeli B, Dietz D, Der-Nigoghossian C, Liyanage-Don N, Rosner GF, Bernstein EJ, Mohan S, Beckley AA, Seres DS, Choueiri TK, Uriel N, Ausiello JC, Accili D, Freedberg DE, Baldwin M, Schwartz A, Brodie D, Garcia CK, Elkind MSV, Connors JM, Bilezikian JP, Landry DW, Wan EY (2021). Post-acute COVID-19 syndrome. Nat Med.

[47] Taquet M, Geddes JR, Husain M, Luciano S, Harrison PJ (2021). 6-month neurological and psychiatric outcomes in 236 379 survivors of COVID-19: a retrospective cohort study using electronic health records. Lancet Psychiat.

[48] Graham EL, Clark JR, Orban ZS, Lim PH, Szymanski AL, Taylor C, DiBiase RM, Jia DT, Balabanov R, Ho SU, Batra A, Liotta EM, Koralnik IJ (2021). Persistent neurologic symptoms and cognitive dysfunction in non-hospitalized Covid-19 "long haulers". Ann Clin Transl Neurol.

[49] Tran V, Porcher R, Pane I, Ravaud P (2022). Course of post COVID-19 disease symptoms over time in the ComPaRe long COVID prospective e-cohort. Nat Commun.

[50] Van Herck M, Goërtz YMJ, Houben-Wilke S, Machado FVC, Meys R, Delbressine JM, Vaes AW, Burtin C, Posthuma R, Franssen FME, Hajian B, Vijlbrief H, Spies Y, van 't Hul AJ, Janssen DJA, Spruit MA (2021). Severe fatigue in long COVID: web-based quantitative follow-up study in members of online long COVID support groups. J Med Internet Res.

[51] Kemp S (2022). Digital 2022: France. Kepios.

[52] (2022). Audience Internet Global en France en décembre 2021. Médiamétrie.

