# Supplementary figures and images for: Identifying Profiles and Symptoms of Patients With Long COVID in France: Data Mining Infodemiology Study Based on Social Media
Source: JMIR Infodemiology. 2022 Nov 22;2(2):e39849. doi: 10.2196/39849 (PMC9685517; doi:10.2196/39849)

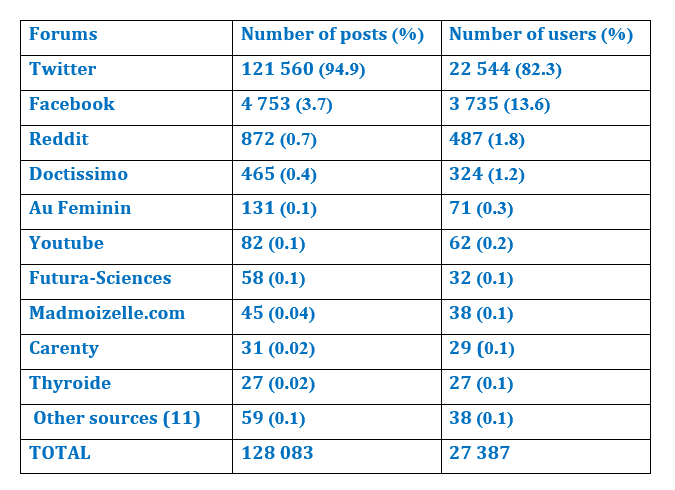

Supplement: Multimedia Appendix 1 [file infodemiology_v2i2e39849_app1.png]

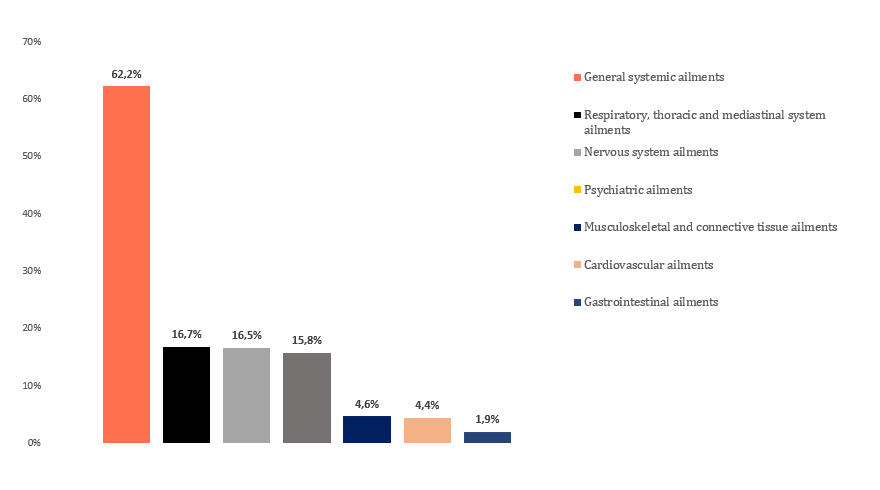

Supplement: Multimedia Appendix 2 [file infodemiology_v2i2e39849_app2.png]

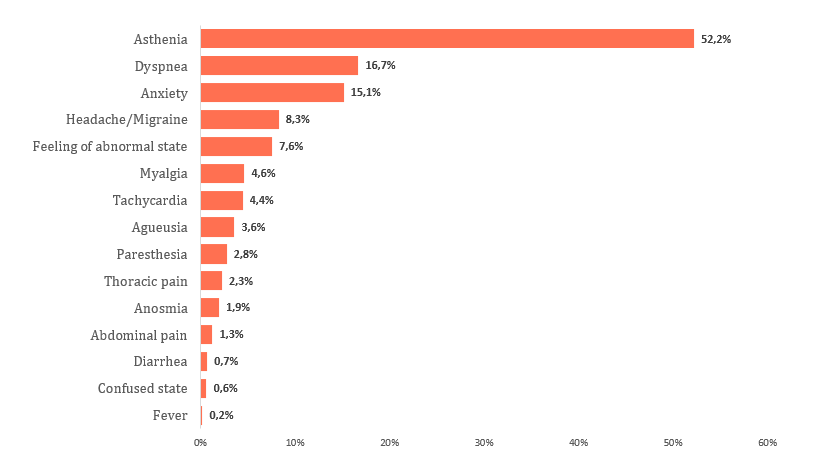

Supplement: Multimedia Appendix 3 [file infodemiology_v2i2e39849_app3.png]
